# Dissimilar effects of stereoisomers and racemic hydroxychloroquine on Ca^2+^ oscillations in human induced pluripotent stem cell‐derived cardiomyocytes

**DOI:** 10.14814/phy2.15760

**Published:** 2023-07-20

**Authors:** Piotr K. Janicki, Amandeep Singh, Arun K. Sharma, Victor Ruiz‐Velasco

**Affiliations:** ^1^ Department of Anesthesiology and Perioperative Medicine Penn State College of Medicine Hershey Pennsylvania USA

**Keywords:** Ca^2+^ oscillations, cardiomyocytes, enantiomers, hydroxychloroquine

## Abstract

All currently employed pharmaceutical formulations of hydroxychloroquine (HCQ) sulfate are a racemate, consisting of equal parts mixture of two stereoisomers: R(−)HCQ and S(+)HCQ sulfates. The aims of the current study were first, to obtain and characterize pure HCQ enantiomers. The separation and purification of free base HCQ enantiomers from the racemate form were performed using semi‐preparative chiral high‐performance liquid chromatography. Second, we compared the pharmacological properties of both optical isomers and racemic mixture on the intracellular Ca^2+^ oscillations employing an in vitro model of human cardiomyocytes derived from induced pluripotent stem cells (iPSCs). The results of the pharmacological investigations indicate that the racemic and pure stereoisomer forms of HCQ sulfate exhibited a dose‐dependent inhibition of spontaneous Ca^2+^ oscillations (as measured from their frequency and Ca^2+^ peak widths) in cardiomyocytes 5–45 min following exposure. In addition, the concentration‐response relationships for all three compounds indicated that the rank order of potency (IC_50_) was R(−)HCQ >racemic HCQ >S(+)HCQ for the frequency of the Ca^2+^ oscillations and width of Ca^2+^ peaks for all time points examined. These studies indicate that both R(−) and S(+) stereoisomers exhibit differing pharmacological actions on hiPSC cardiomyocytes, with the former effecting a greater potency on cell Ca^2+^ handling.

## 1 INTRODUCTION

The current COVID‐19 pandemic brought several older drugs back to the spotlight because of their potential antiviral activity. One of the most controversial drugs from this group includes hydroxychloroquine (HCQ) which had been recognized for several years as an effective agent in treating malaria, rheumatoid arthritis, and other autoimmune disorders (Schrezenmeier & Dörner, 2020; Shukla & Wagle, 2019). However, after HCQ began to be employed in the prophylaxis and/or treatment of COVID‐19 in a larger number of patients, its use had been reported to be associated with significant pro‐arrhythmic activity (Bauman & Tisdale, 2020; Oscanoa et al., 2020; Sinha & Balayla, 2020). This was more apparent when administered with other QT prolonging drugs or taken in higher doses when compared to that of the typical anti‐malaria dosage. Although these adverse effects could be multifactorial, they are most likely associated with the block of several voltage‐gated ion channels. These include both the delayed rectifier K^+^ channels (hERG or K_IR_) coded by the ether a‐gogo‐related gene and Kir2.1 potassium channel (product of KCJN2 gene) (Tamargo et al., 2004). Other channels that could be targeted by HCQ include voltage‐gated Ca^2+^ (Ca_V_1.2), Na^+^ (Na_V_1.5), and K^+^ (K_V_4.3 and K_V_7.1) channels, albeit at higher concentrations (Ballet et al., 2022; Capel et al., 2015; Grilo et al., 2010; Okada et al., 2020). Additionally, Capel et al. (2015) reported that HCQ blocked the pacemaker currents (I_f_, “funny” current) in isolated guinea pig myocytes.

All currently used pharmaceutical formulations of HCQ sulfate are a racemate, consisting of approximately equal parts mixture of two stereoisomers: S(+)HCQ and R(−)HCQ sulfates. It has been demonstrated previously that chirality can influence the pharmacokinetic and pharmacodynamic properties of HCQ enantiomers (Brocks & Mehvar, 2003; Ducharme et al., 1994; Jia et al., 2022; Miller & Ulrich, 2008; Tett et al., 1994; Wainer et al., 1994), but to a lesser degree with the latter. There is a scarcity of information detailing whether the HCQ enantiomers exhibit differences regarding their effects on cardiac muscle given their differing pharmacological block of K^+^ channels resulting from their enantiomeric nature (Ballet et al., 2022; Li et al., 2020). If, in fact, such difference could be established, then the less cardiotoxic enantiomer would be a more likely suitable and effective drug when used as the sole agent in the pharmaceutical formulations of HCQ, when compared with the current racemic formulations as previously described (D'Acquarica & Agranat, 2020; Lentini et al., 2020).

Human induced pluripotent stem cell (iPSC)‐derived in vitro model systems have recently emerged as a physiologically relevant and highly reproducible option for testing cardiomyocyte Ca^2+^ handling properties in drug development (Burnett et al., 2021; Satsuka & Kanda, 2020). The iPSC‐derived cardiomyocytes are a particularly attractive in vitro model as they form a synchronously beating monolayer that can be used to reliably reproduce drug‐associated cardiac physiology phenotypes using a fast‐kinetic fluorescence assay capable of monitoring of Ca^2+^ changes (Sirenko, Crittenden, et al., 2013; Sirenko, Cromwell, et al., 2013). Of particular interest is the ability to employ this cardiomyocyte cell type to test for the potential of several compounds to induce cardiac arrhythmias as a result of interfering with cardiomyocyte repolarization, that is, the in vitro equivalent to clinical QT prolongation. Therefore, the main aims of the current study were, first, to obtain and characterize pure forms of HCQ enantiomers. Second, we compared the pharmacological properties of these optical isomers using this in vitro human cell system.

## 2 MATERIALS AND METHODS

### 2.1 Preparation of free base racemic HCQ

HCQ sulfate powder (racemate form) was purchased from AK Scientific, Inc., (Union City, CA). Prior to the separation of the optical isomers, HCQ sulfate was converted into the free base. In short, a 10% NaOH solution was added dropwise to a stirred solution of racemic HCQ sulfate (1.0 g, 2.3 mmol) in 10 mL of H_2_O at 0°C and the reaction mixture was stirred for 1 h at room temperature. The formed free base HCQ racemate was then extracted with ethyl acetate (3 × 15 mL). The combined organic extracts were dried with sodium sulfate and concentrated to obtain the free base HCQ racemate oil (750 mg) which had a pale‐yellow appearance. This product was used for chiral chromatography separation without further purification.

### 2.2 Separation of HCQ enantiomers by chiral high‐performance liquid chromatography (HPLC)

Separation of both optical isomers from racemic sulfate‐free HCQ was achieved using chiral HPLC. We first employed an analytical column (Enantiocel^R^ G1‐5, 250 × 4.6 mm) to establish and optimize the separation conditions. To obtain larger amounts (i.e., >1.0 g), the Enantiocel G1‐5 semi‐preparative HPLC column (250 × 10 mm, 5 μm) was employed. Both columns were obtained from ColumnTek, LLC (State College, PA). The isocratic HPLC system consisted of Water 410 pump, Rheodyne manual loop injector, and multi‐length Water detector at 240 nm. The mobile phase consisted of tert‐butylmethyl‐ether and ethanol (HPLC grade, both from Sigma‐Aldrich Inc.) at a ratio (v/v) of 90:10 with the addition of 0.2% of diethylamine (HPLC grade, Sigma‐Aldrich Inc.). The flow rate was 1 mL/min for the analytical column and 4 mL/min for the preparative column at room temperature. The fractions corresponding to two distinct HPLC peaks were collected and stored at −20°C. After combining fractions from several HPLC runs, the HPLC phase was evaporated with a rotary evaporator to an amorphous, semi‐solid substance for both fractions consisting of a free base of pure HCQ isomers.

In our initial experiments, it was determined that the amorphous material was not suitable for the synthesis of HCQ sulfate. Therefore, the product was cleaned by adding water, which caused precipitation of water‐insoluble precipitate that was subsequently diluted in pure ethanol. The concentrated sulfuric acid (27 mg, 1 mmol) was added at 0°C of optically pure HCQ R(−) or S(+) enantiomer (100 mg, 0.28 mmols) solution in absolute ethanol, and the reaction mixture was stirred for 12 h at room temperature. After complete conversion, the precipitates of corresponding HCQ salts were filtered off and washed with diethyl ether to yield the corresponding optically pure HCQ sulfate (100 mg) as a white solid. The analysis of both enantiomers (R(−)‐ and S(+)‐HCQ sulfate) was performed using both analytical HPLC on Enantiocel column, magnetic resonance spectroscopy, and circular dichroism (CD) spectra to identify the purity of enantiomers.

### 2.3 Cardiomyocyte cultures

Human iPSC‐derived cardiomyocytes were obtained from Caucasian donor cells (gender of the donor was not provided by manufacturer of the cells) with no known disease‐related (iCell™ cardiomyocytes, FujiFilm Cellular Dynamics International). Both plating and maintenance media were also provided by FujiFilm Cellular Dynamics. Cardiomyocytes were plated and maintained according to the manufacturer's recommendations as described previously (Sirenko, Crittenden, et al., 2013). Cardiomyocytes were plated onto 0.1% gelatin coated 96 multi‐well plates wells at a density of 50,000 cells/well in maintenance media (final volume, 200 μL). The cardiomyocytes were stored in a humidified incubator (5% CO_2_/95% air) at 37°C for 10 days. The well media were replaced every 48 h. Synchronous beating of the cardiomyocytes was evident 5–7 days after plating. Fluorescence experiments were performed 10 days after plating. At this time, the cardiomyocytes demonstrated regular synchronous beating (Video S1, Control Cardiomyocytes Play Speedx4.mp4 [figshare.com]).

### 2.4 Measurement of intracellular Ca^2+^ fluxes

In this set of experiments, Ca^2+^ ion flux assay was initially optimized for the high throughput screening in 96‐well plates using the FlexStation 3 multi‐mode microplate reader (37°C) and EarlyTox Cardiotoxicity Kit (both from Molecular Devices). We measured the HCQ‐mediated changes in both the concentration‐dependent widening of Ca^2+^ peaks and in the cell‐beating of the cardiomyocytes. On the day of experiment, the cells were loaded with Ca^2+^ indicator dye (CardioTox) for 1 h with a final volume of 100 μL at 37°C. Prior to exposure to the HCQ compounds, baseline (control) Ca^2+^ levels were first acquired. Thereafter, the plate reader's robotic arm system applied the compound of interest. Only one concentration per well was performed. Fluorescence readings were acquired 5, 15, 30, and 45 min following application of the compounds. Fluorescence measurements of the CardioTox dye (excitation at 485 nm, emission at 535 nm) were acquired at about three frames per sec (3 Hz), 0.388 s interval for three wells simultaneously, and up to 78 frames per well were collected during a total period of 30 sec. The selected sampling rate of the continuous fluorescence signal exceeded the Nyquist frequency (1.5 Hz) calculated for the highest expected Ca^2+^ oscillation frequency observed in the cardiomyocytes (20/min or 0.33 Hz). Based on Nyquist theorem, the sampling rate exceeding Nyquist frequency is required for presumed maximum frequency of the sample signal provided that a sampling rate was performed at a sufficiently high rate. The plates were returned to the incubator between fluorescence measurements. In some cases, we calculated the concentration required for complete cessation of the Ca^2+^ oscillations.

### 2.5 Data analysis

The primary outcome for the analyzed data was represented by peak Ca^2+^ oscillations frequency (i.e., beats per minute) and width of the recorded Ca^2+^ peak at 10% of the maximum amplitude, evaluated during at least a continuous 30 s period of recording during selected time points after drug administration. The HCQ concentration‐response curves for each HCQ compound were fit to the Hill equation: *I* = *I*_MAX_/[1 + (IC_50_/[compound])^nH^], where *I* is the % inhibition, *I*_MAX_ is the maximum inhibition of the compound, IC_50_ is the half‐inhibition concentration, [compound] is the HCQ concentration and nH is the Hill coefficient. The IC_50_ value was calculated separately for each HCQ sulfate isomer (or racemate) and both outcome parameters recorded and represent drug concentration (in M) at which 50% decrease in the beat frequency, maximum amplitude, and peak width occur.

### 2.6 Statistical analysis

For statistical analysis of the non‐linear fits of averaged (over 30 s observation period) measured parameters versus log of compound concentrations were determined by extra sum‐of‐squares *F* test for nested models employing statistical calculator from Prism version 9.5 (GraphPad). In addition, the statistical significance of differences between inhibitory effects of investigated compounds on Ca^2+^ oscillations at different time points were analyzed using independent ANOVA test.

## 3 RESULTS

**FIGURE 1:**
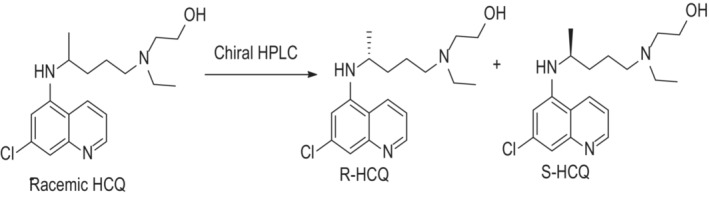
Schematic representation of chemical structure and the separation of racemic hydroxychloroquine (HCQ) into its optical isomers.

**FIGURE 2:**
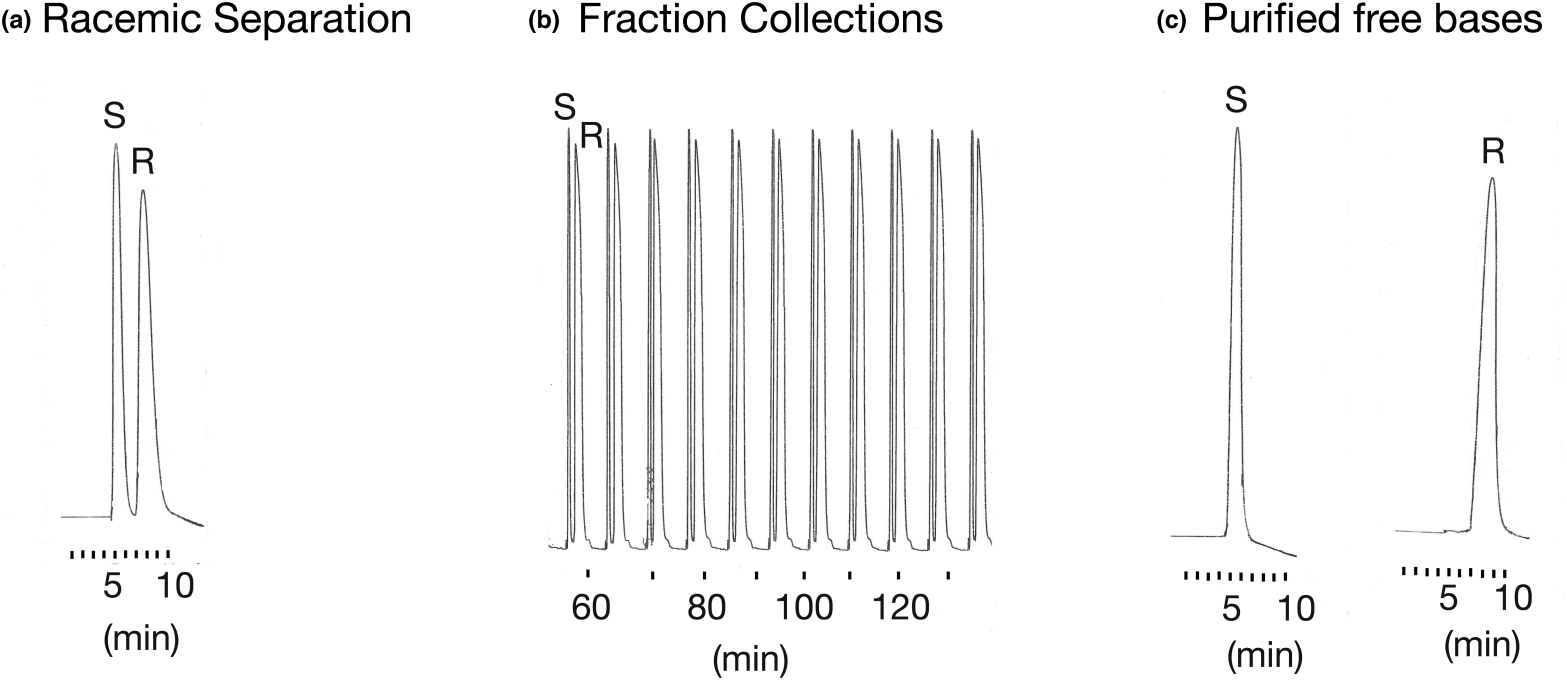
Chiral high‐performance liquid chromatography (HPLC) separation of free base racemic hydroxychloroquine (HCQ) on the analytical HPLC column (a), serial injections used for fraction collections on semi‐preparative HPLC column (b), and HPLC image of purified free bases of R(−)HCQ and S(+)HCQ after injection of pure optical isomers onto analytical chiral HPLC column (c). Note that the recordings were performed with different amplification of the signal and recording speed.

**FIGURE 3:**
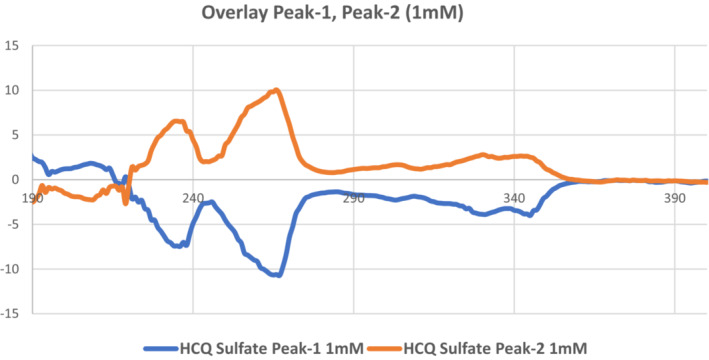
Circular dichroism (CD) spectra of R(−)hydroxychloroquine (HCQ) (2nd—peak on high‐performance liquid chromatography (HPLC) separation, orange trace) and S(+)HCQ sulfate (first peak on HPLC separation, blue trace). Data are presented as molecular ellipticity difference (delta epsilon in M^−1^ cm^−1^) for 1 mM concentrations of stereoisomers in water versus wavelength (nm) (for details of the method see Okuom et al., 2015). The R(−) enantiomer has a positive cotton effect and the S(+) enantiomer has a negative cotton effect centered between 200 and 290 nm.

The separation and purification of free base HCQ enantiomers from the racemic form were performed using semi‐preparative chiral HPLC (Figure 1). In the first set of experiments, we employed an analytical chiral HPLC column to separate the racemic compound in order to collect the stereoisomers. The results from this trial separation, as shown in Figure 2a, indicate that we could successfully obtain both HCQ enantiomers. We next employed a semi‐preparative column that would yield larger quantities (i.e., greater than 1000 mg) of both free base R(−) and S(+)HCQ. The plot shown in Figure 2b indicates that we were successful in obtaining sufficient quantities of both enantiomers. Athough the peak height for the S(+) compound was taller than that obtained for the R(−) enantiomer, the latter exhibited a wider peak, suggesting that the area under the curve (AUC) for both species would be comparable. Thereafter, each enantiomer compound was transformed into crystalline, water‐soluble sulfate salts. The analysis of the chiral identity and purity of the HCQ stereoisomers were obtained using analytical chiral HPLC of each fraction (Figure 2c), NMR spectra, and CD spectra (Figure 3). The results from these three processes indicated that the purity of the isolated stereoisomers exceeded 99.5%.

In the next set of experiments, cardiomyocyte contraction rate and pattern were characterized by measuring changes in intracellular Ca^2+^ measured with the Ca^2+^ sensitive dye. In the initial experiments, we found that HCQ concentrations greater than 300 μM led to extreme and erratic changes in Ca^2+^ levels within seconds of exposure (data not shown). Video S2 shows contraction and relaxation of cardiomyocytes 5 min after exposure to 400 μM racemic HCQ (Plus HCQ 0.4 mM Play Speedx4.mp4 [figshare.com]). Full concordance between mechanical contraction and Ca^2+^ signal was demonstrated by adding a high dose of HCQ, which inhibited completely the Ca^2+^ oscillations and cardiomyocyte contractions in an identical period. Further, these effects were irreversible and, consequently, we did not employ HCQ concentrations greater than 300 μM.

**FIGURE 4:**
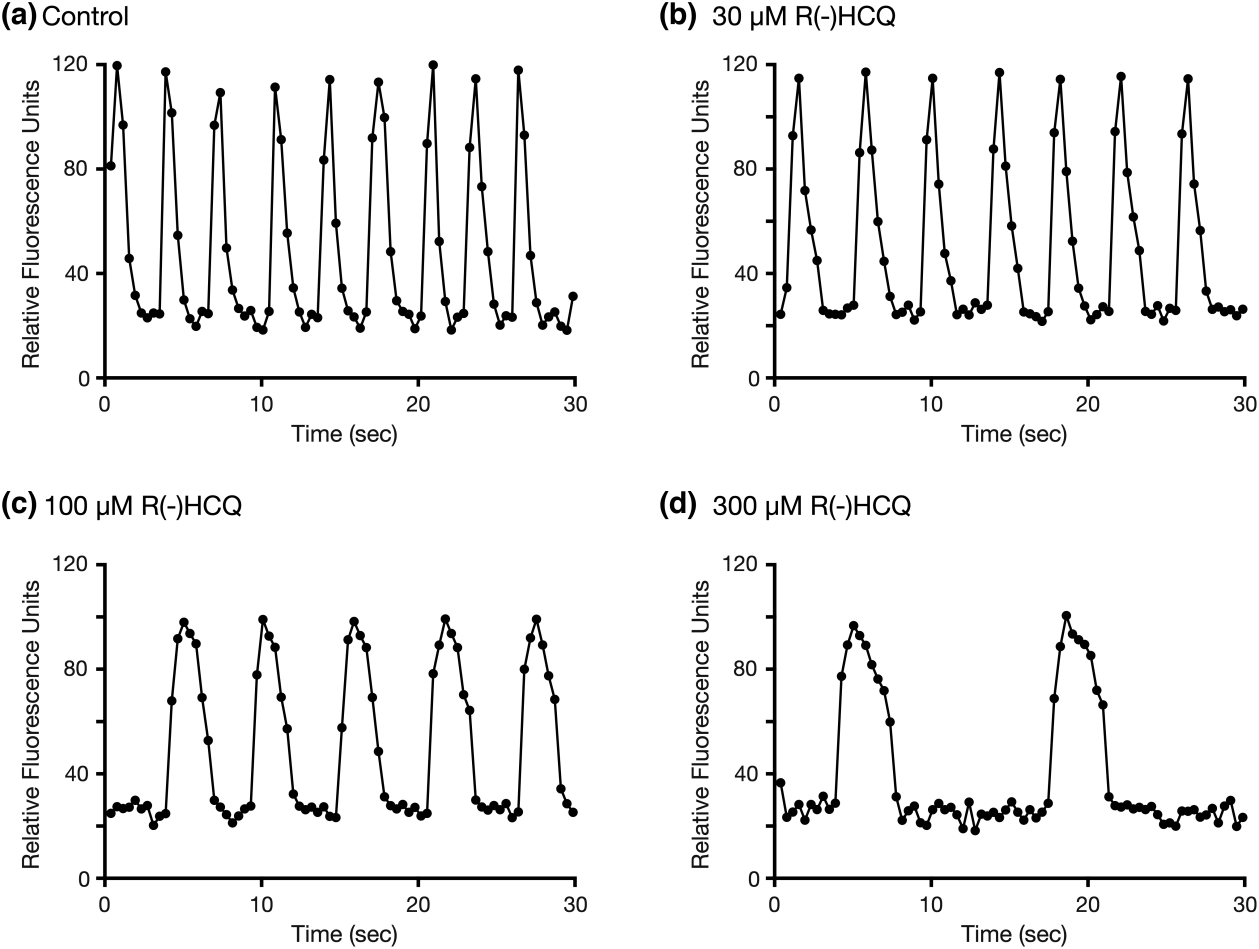
Effect of R(−)hydroxychloroquine (HCQ) on Ca^2+^ oscillations and Ca^2+^ wave width. Sample baseline Ca^2+^ oscillations acquired from a single microplate well in human induced pluripotent stem cell (iPSC)‐derived cardiomyocytes 5 min after the application of control buffer (a), 30 μM R(−)HCQ (b), 100 μM R(−)HCQ (c) and 300 μM R(−)HCQ (d). Note that oscillation frequency decreased, and wave width increased with HCQ concentrations greater than 100 μM.

Figure 4a shows representative recording of the baseline (control) spontaneous Ca^2+^ oscillations in cardiomyocytes prior to exposure to HCQ compounds. The frequency of these oscillations ranged from 17 to 23 waves (or beats) per min (18.9 ± 2.0, mean ± SD, *n* = 3). Following the addition of the control buffer, we did not observe overt changes in the frequency during the entire recording period (45 min). The exposure of the cardiomyocytes to 30 μM R(−)HCQ led to a decrease in the number of Ca^2+^ oscillations (Figure 4b), while the application of 100 μM R(−)HCQ resulted in a further diminution of the oscillations and widening of the Ca^2+^ peaks (Figure 4c) 5 min post‐exposure of the HCQ isomer. The trace in Figure 4d illustrates that exposure to 300 μM R(−)HCQ resulted in further widening of the Ca^2+^ peaks and decrease in oscillations 5 min after the application of HCQ.

**FIGURE 5:**
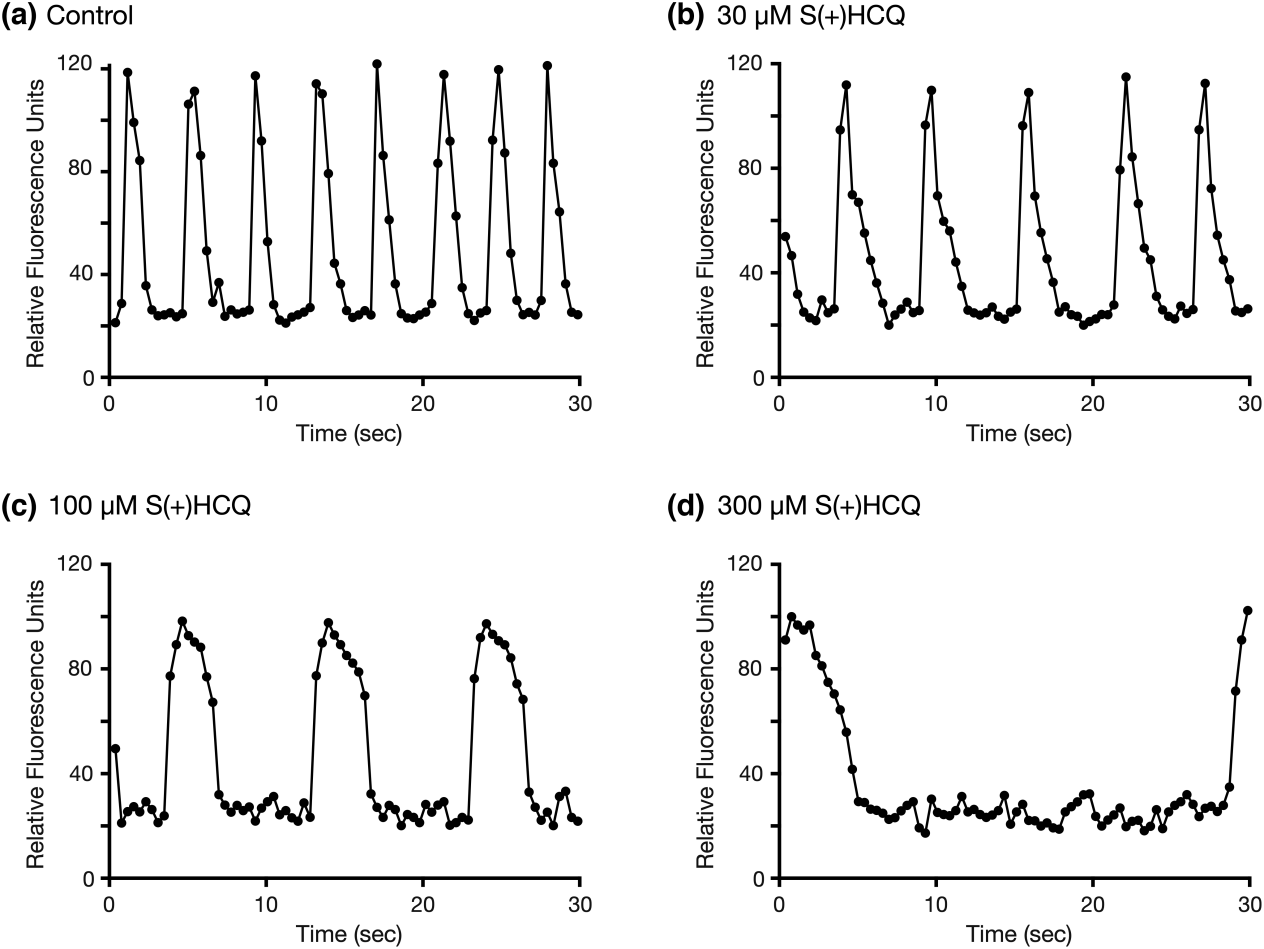
Effect of S(+)hydroxychloroquine (HCQ) on Ca^2+^ oscillations and Ca^2+^ wave width. Sample baseline Ca^2+^ oscillations acquired from a single microplate well in human induced pluripotent stem cell (iPSC)‐derived cardiomyocytes 5 min after the application of control buffer (a), 30 μM S(+)HCQ (b), 100 μM S(+)HCQ (c) and 300 μM S(+)HCQ (d). Note that oscillation frequency decreased, and wave width increased with HCQ concentrations greater than 100 μM.

**FIGURE 6:**
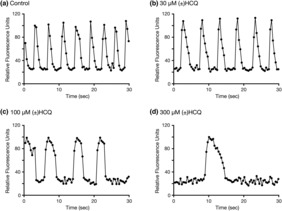
Effect of (±)hydroxychloroquine (HCQ) racemate on Ca^2+^ oscillations and Ca^2+^ wave width. Sample baseline Ca^2+^ oscillations acquired from a single microplate well in human induced pluripotent stem cell (iPSC)‐derived cardiomyocytes 5 min after the application of control buffer (a), 30 μM (±)HCQ (b), 100 μM (±)HCQ (c) and 300 μM (±)HCQ (d). Note that oscillation frequency decreased, and wave width increased with HCQ concentrations greater than 100 μM.

**FIGURE 7:**
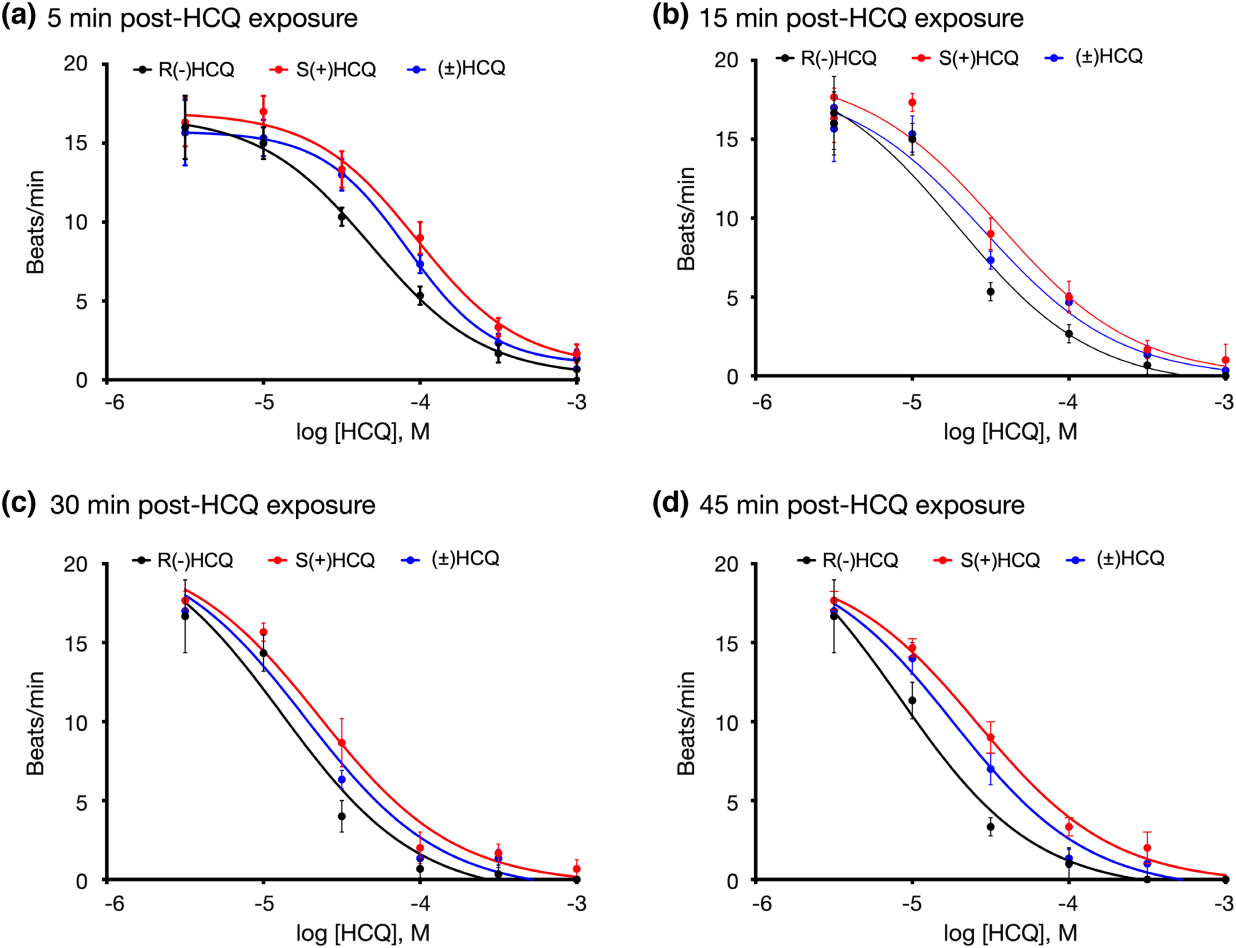
Racemate and enantiomeric hydroxychloroquine (HCQ) concentration‐response relationships of human cardiomyocytes 5‐ (a), 15‐ (b), 30‐ (c), and 45 min (d) following HCQ exposure. Each data point represents the (mean ± SD) oscillation frequency (beats/min) from three independent experiments with 6–8 wells per concentration. The smooth curves were obtained by fitting the data points to the Hill equation. The IC_50_ values are listed in Table 1.

**FIGURE 8:**
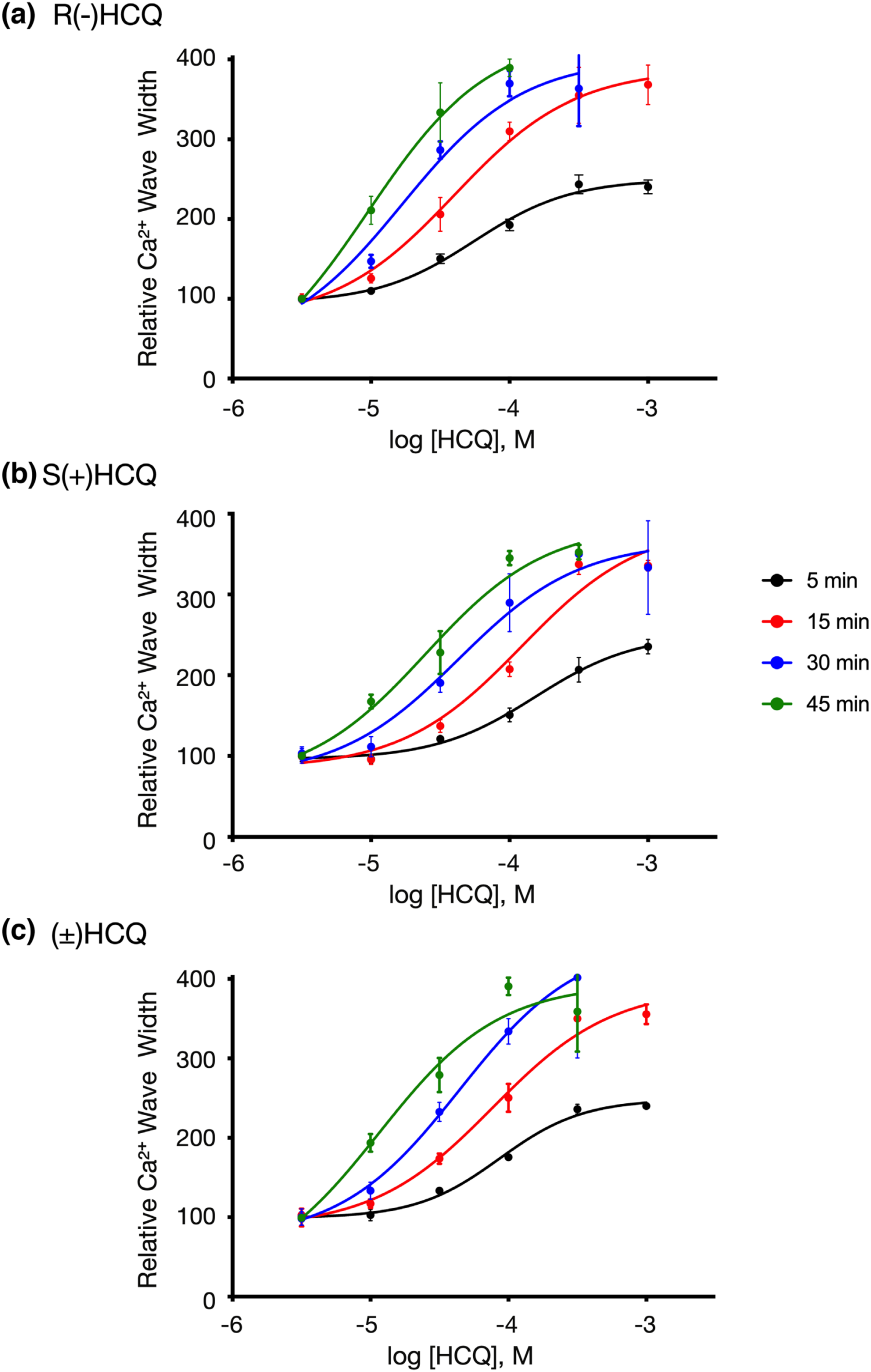
Racemate and enantiomeric hydroxychloroquine (HCQ) concentration‐response relationships of human cardiomyocyte Ca^2+^ peak width for R(−)HCQ (a), S(+)HCQ (b), and ±HCQ compounds. The plots in each panel denote the (mean ± SD) Ca^2+^ peak width 5‐, 15‐, 30‐, and 45 min after HCQ compound application. Each data point was acquired from three independent experiments with 6–8 wells per concentration. The smooth curves were obtained by fitting the data points to the Hill equation. The IC_50_ values are listed in Table 2.

Figures 5a–d and 6a–d show representative examples of recordings acquired in cardiomyocytes after application of the HCQ enantiomers and racemic HCQ. Both compounds exerted similar effects on both Ca^2+^ oscillations and Ca^2+^ wave width as that obtained with R(−)HCQ. The R(−)HCQ concentration‐response curves at 5‐, 15‐, 30‐, and 45‐min post‐isomer exposure for the frequency of Ca^2+^ oscillations and Ca^2+^ wave widths are plotted in Figures 7a, 5d, 8a, and 6c, respectively. The IC_50_ values for both parameters are listed in Tables 1 and 2. It can be observed that the R(−)HCQ isomer exerted the highest effect on both parameters 45 min post‐compound exposure with an IC_50_ of 8.3 μM and 9.5 μM for the Ca^2+^ oscillations and Ca^2+^ wave width, respectively. Figure 8 also illustrates that there was a time‐dependent leftward shift of the fits resulting from increased Ca^2+^ wave width. The absolute values of the Ca^2+^ oscillation frequency are shown in Tables 3, 4, 5, 6 at times following the exposure of the cells to the compounds. The absolute values for each compound concentration were compared using one‐way ANOVA test and *p* < 0.05 was considered statistically significant. The results indicate that the oscillation frequency decreased significantly for in a time‐ and concentration‐dependent manner.

**TABLE 1:** IC_50_ values for frequency of Ca^2+^ oscillation response calculated from concentration‐response curves for both responses (Ca^2+^ peak width and frequency of Ca^2+^ oscillations). Statistical comparison of dose–response curves indicate that all curves were different within each dose–response group at time intervals at *p* < 0.001 (extra sum‐of‐squares *F* test).

Time of recording post‐compound exposure	R(−)HCQ (M)	S(+)HCQ (M)	(±)HCQ (M)
5 min	4.89 × 10^−5^	9.51 × 10^−5^	8.23 × 10^−5^
15 min	1.93 × 10^−5^	3.51 × 10^−5^	2.83 × 10^−5^
30 min	1.31 × 10^−5^	2.26 × 10^−5^	1.85 × 10^−5^
45 min	0.83 × 10^−5^	2.61 × 10^−5^	1.83 × 10^−5^

**TABLE 2:** IC_50_ values for Ca^2+^ peak width response calculated from concentration‐response curves for both responses (Ca^2+^ peak width and frequency of Ca^2+^ oscillations). Statistical comparison of dose–response curves indicate that all curves were different within each dose–response group at time intervals at *p* < 0.001 (extra sum‐of‐squares *F* test).

Time of recording post‐compound exposure	R(−)HCQ (M)	S(+)HCQ (M)	(±)HCQ (M)
5 min	5.65 × 10^−5^	15.37 × 10^−5^	8.61 × 10^−5^
15 min	4.01 × 10^−5^	12.21 × 10^−5^	7.68 × 10^−5^
30 min	1.67 × 10^−5^	4.28 × 10^−5^	4.1 × 10^−5^
45 min	0.95 × 10^−5^	2.47 × 10^−5^	1.15 × 10^−5^

**TABLE 3:** Effect of different optic isomers of hydroxychloroquine (HCQ) or racemate on the Ca^2+^ oscillation frequency in human cardiomyocytes 5 min after administration (*n* = 3 independent experiments). One‐way ANOVA analysis of frequency values obtained for each isomer (*p* < 0.05).

Concentration (log M)	R(−)HCQ	S(+)HCQ	Racemic HCQ	*p*‐Value
	Oscillations/min mean ± SD	Oscillations/min mean ± SD	Oscillations/min mean ± SD	
Control	19.0 ± 2.0	18.6 ± 2.0	19.3 ± 2.0	0.89
−5.0	15.0 ± 1.0	17.0 ± 1.0	15.3 ± 1.1	0.11
−4.5	10.3 ± 0.5	13.3 ± 1.1	13.0 ± 1.0	0.01
−4.0	5.3 ± 0.5	9.0 ± 1.0	7.3 ± 0.5	0.002
−3.5	1.7 ± 0.5	3.3 ± 0.5	2.3 ± 0.5	0.03
−3.0	0.7 ± 0.5	1.7 ± 0.5	1.3 ± 0.5	0.17

**TABLE 4:** Effect of different optic isomers of hydroxychloroquine (HCQ) or racemate on the Ca^2+^ oscillation frequency in human cardiomyocytes 15 min after administration (*n* = 3 independent experiments). One‐way ANOVA analysis of frequency values obtained for each isomer (*p* < 0.05).

Concentration (log M)	R(−)HCQ	S(+)HCQ	Racemic HCQ	*p*‐Value
	Oscillations/min mean ± SD	Oscillations/min mean ± SD	Oscillations/min mean ± SD	
Control	19.0 ± 2.0	18.6 ± 2	19.3 ± 2.0	0.89
−5.0	16.6 ± 2.3	17.6 ± 0.5	18.1 ± 1	0.5
−4.5	15.3 ± 0.5	9 ± 1.0	7.3 ± 0.5	0.002
−4.0	2.6 ± 0.5	5 ± 1.0	4.6 ± 0.5	0.01
−3.5	0.6 ± 0.5	1.6 ± 0.5	1.3 ± 0.5	0.1
−3.0	0	1.1 ± 1	0.3 ± 0.5	NC

*Note*: NC – statistical significance not calculated.

**TABLE 5:** Effect of different optic isomers of hydroxychloroquine (HCQ) or racemate on the Ca^2+^ oscillation frequency in human cardiomyocytes 30 min after administration (*n* = 3 independent experiments). One‐way ANOVA analysis of frequency values obtained for each isomer (*p* < 0.05).

Concentration (log M)	R(−)HCQ	S(+)HCQ	Racemic HCQ	*p*‐Value
	Oscillations/min mean ± SD	Oscillations/min mean ± SD	Oscillations/min Mean ± SD	
control	19.0 ± 2.0	18.6 ± 2.0	19.3 ± 2.0	0.89
−5.0	14.3 ± 1.1	15.6 ± 0.5	15.0 ± 1.0	0.2
−4.5	4.1 ± 1.0	8.6 ± 1.5	6.3 ± 0.5	0.006
−4.0	0.6 ± 0.5	2.6 ± 2.0	1.0 ± 1.0	0.05
−3.5	0.3 ± 0.5	3.3 ± 0.5	2.3 ± 0.5	0.04
−3.0	0	0.33 ± 0.3	0	NC

*Note*: NC – statistical significance not calculated.

**TABLE 6:** Effect of different optic isomers of hydroxychloroquine (HCQ) or racemate on the Ca^2+^ oscillation frequency in human cardiomyocytes 45 min after administration (*n* = 3 independent experiments). One‐way ANOVA analysis of frequency values obtained for each isomer (*p* < 0.05).

Concentration (log M)	R(−)HCQ	S(+)HCQ	Racemic HCQ	*p*‐Value
	Oscillations/min Mean ± SD	Oscillations/min Mean ± SD	Oscillations/min Mean ± SD	
control	19.0 ± 2.0	18.6 ± 2.0	19.3 ± 2.0	0.89
−5.0	11.3 ± 1.1	14.6 ± 0.5	14.0 ± 1.0	0.01
−4.5	3.3 ± 0.5	9.0 ± 1	7.0 ± 1	0.0006
−4.0	1 ± 1.0	2.3 ± 0.5	1.3 ± 0.5	0.01
−3.5	0	2.1	1.0 ± 1.0	NC
−3.0	0	0	0	NC

*Note*: NC – statistical significance not calculated.

## 4 DISCUSSION

There are several known ways of obtaining pure optical isomers of HCQ. The synthetic one, patented in 1994 (US Patent 5,314,894 by Blaney et al., 1994; Stecher et al., 1994), is relatively time‐consuming, expensive, requires specialized chemical laboratory, and is more suitable for the synthesis of a larger amount of HCQ. There have been also described some new pathways of stereo HCQ synthesis, which possibly can make the synthetic process easier (Ni et al., 2022). We also attempted to obtain pure isomers of HCQ using previously described kinetic resolution of racemic HCQ (Craiga & Ansari, 1993); however, the obtained optical isomers were not sufficiently pure for use in subsequent pharmacological studies. We used, consequently, chiral HPLC columns to separate isomers after injection of racemic HCQ free base, similar to methods described previously (Ballet et al., 2022; Li et al., 2020; Xiong et al., 2021). The resolution of racemic HCQ into two optical isomers was complete and of excellent quality from both analytical and semi‐preparative columns, which allowed for the collection of fractions from both chromatographic peaks for further synthesis of HCQ sulfate enantiomers. The results of further chemical analysis of both isomers indicated that both isomers exhibited >99.5% purity and were suitable for pharmacological testing in cardiomyocytes. In addition, we were able to design a method of conversion of the lipid‐soluble HPLC output into water‐soluble and crystalline sulfate salts.

The results of pharmacological investigations showed that HCQ sulfate (both racemate and stereoisomers) produced a dose‐dependent inhibition of spontaneous Ca^2+^ oscillations in human cardiomyocytes over the investigated time of 5–45 min (i.e., a decrease in Ca^2+^ oscillation frequency and an increase in the width of Ca^2+^ peaks). In addition, based on the results of the dose–response analysis, the potency of HCQ to inhibit these oscillations was dependent on the optical configuration of HCQ, with a “pro‐arrhythmic” potency magnitude of R(−)HCQ >racemic HCQ >S(+)HCQ. Identical potency differences were obtained for all methods evaluating Ca^2+^ oscillations frequency and width of Ca^2+^ peaks, as well as all investigated time intervals. The longer time of incubation with HCQ resulted in increased inhibitory effects on Ca^2+^ oscillations. Based on the obtained results, it can be concluded that the Ca^2+^ oscillations reflect pro‐arrhythmic effect of different optical formulations of HCQ and that S(+)HCQ represent less pro‐arrhythmic potential compared with R(−)HCQ, with racemic formulations representing intermediate potency.

The present study aimed to compare the cardiotoxic properties of HCQ enantiomers without further dissecting its molecular basis. Drug‐induced long QT syndrome is an established cardiac side effect of a wide range of medications and represents a significant concern for drug safety. The rapidly and slowly activating delayed rectifier K^+^ currents, mediated by channels encoded by the human ether‐a‐go‐go–related gene (*hERG*) and inwardly rectifying Kir2.1, are two main K^+^ currents responsible for ventricular repolarization. Several recent studies employing HEK cells or human‐derived cardiomyocytes (Ballet et al., 2022; Thomet et al., 2021; Wang et al., 2021) have demonstrated block by HCQ at μM concentration range of both of these K^+^ channels. However, the block of Kir2.1 currents was greater (i.e., lower potency) than that observed for hERG channel currents (Ballet et al., 2022). Like the present study, the R(−)HCQ enantiomer exerted a more potent inhibition than S(+)HCQ for both hERG and Kir2.1 channels, with an enantiomeric separation of 2‐ to 4‐fold (Ballet et al., 2022). In subsequent experiments in the rabbit Purkinje fibers, the authors demonstrated different electrophysiological properties for both HCQ enantiomers, with R(−)HCQ prominently depolarizing the membrane resting potential and inducing autogenic activity, while S(+)HCQ primarily increased the action potential duration. Nevertheless, the authors concluded that the chirality of HCQ does not substantially influence the arrhythmogenic properties in vitro. The results from the current study indicate that with a similar HCQ concentration range and similar enantiomeric separation (2‐ to 4‐fold), both enantiomers of HCQ (R>S) produced significant pro‐arrhythmic activity by altering the Ca^2+^ oscillations in human cardiomyocytes, commonly known accepted as the model for pre‐clinical studies of potentially pro‐arrhythmic drugs. One explanation for this difference could be that Ballet et al. (2022) observed single channel data, whereas in our model we registered a more complex, end‐effect (i.e., Ca^2+^ oscillations) which includes HCQ‐induced alterations of many channels in cardiomyocytes. In addition, their data on Purkinje cells were obtained using the rabbit model which may display some interspecies differences from our human cardiac cells. It should also be mentioned that another report lent support to the assumption that the S(+)HCQ enantiomer is less potent than the R(+) enantiomer in blocking hERG channels in transfected cells (Li et al., 2020). Although no effects of HCQ (up to 90 μM concentrations) were observed for Na_V_1.5, Ca_V_1.2, K_V_4.3 or K_V_7.1 channels (Ballet et al., 2022), both Thomet et al. (2021) and Wang et al. (2021) observed block of these channels by HCQ. The latter two studies did not indicate whether HCQ was racemic. Also, experimental conditions may help explain this discrepancy.

The results obtained in this study should be also discussed with respect to previously described data about circulating levels of HCQ after administration of therapeutic doses (i.e., 400 mg daily PO). The previously reported average HCQ plasma concentrations were in the range 0.5–1 μM (Carlsson et al., 2020). The in vitro plasma protein binding studies of HCQ enantiomers indicate that the free fraction of R(−)HCQ (i.e., responsible for the pharmacodynamic effect of HCQ) was about two times higher compared to S(+)HCQ, at 63% and 36%, respectively (McLachlan et al., 1993). The described data clearly indicate that the reported therapeutic plasma concentrations of HCQ are in sub‐micromolar range in humans, and thus well below concentrations of HCQ effective in our in vitro model, in which concentrations of either HCQ enantiomers or its racemate did not produce any effects on Ca^2+^ oscillations at <10 μM. It should be mentioned, however, that in specific circumstances (e.g., doses of HCQ >400 mg daily, long QT syndrome, electrolyte imbalance, concomitant use of drugs prolonging QT interval), HCQ and/or enantiomers can produce pro‐arrhythmic effects at a lower concentration range. Based on our results, in such situations, the therapeutic use of S(+)HCQ could be more beneficial than the use of racemate compound.

In addition, the therapeutic use of HCQ in doses higher than those prescribed for antimalaria prophylaxis/treatment was advocated based on recent data obtained from several clinical trials. Although the use of HCQ remains a questionable topic for the scientific community, based on their ability to suppress in vitro replication of several coronaviruses, it is nevertheless clinically employed to treat COVID‐19 in some countries due to the lack of availability of more adequate medication. The hypothesis that HCQ could enhance patients' clinical outcomes with SARS‐CoV‐2 is confirmed. Few reports indicate that it has some activity against viral infection (Das et al., 2021). If this is the case, the availability and therapeutic use of S(+)HCQ instead of racemic (±)HCQ could be beneficial from the safety point of view. This hypothesis should be, however, confirmed in further clinical studies comparing pharmacological activity and side effects associated with the use of HCQ racemate and its S(+) enantiomer. This seems to be particularly important in light of some recent and still controversial observations indicating in vitro differences in anti‐COVID virus efficiency between HCQ enantiomers (Li et al., 2020; Ni et al., 2022).

Although the primary goal of the study was to compare the pro‐arrhythmic effects of enantiomers of HCQ in human iPSC cardiomyocytes, there are some limitations. That is, we did not examine the mechanism (i.e., ion channel activity) of the observed differences in the pharmacological profile of the HCQ enantiomers. The identification of the actual cell surface target could be further explored in the subsequent studies. Another limitation of the study has been the selection of only one method (i.e., Ca^2+^ sensitive fluorescent dye) for the observed effects of HCQ enantiomers on the Ca^2+^ handling in the cardiomyocytes. The other limitation of the study involves rather slow capturing rate of the Ca^2+^ oscillations.

In summary, we found a significant degree of enantiomeric separation between R(−) HCQ and S(+)HCQ in their pro‐arrhythmic activity on Ca^2+^ fluxes in a human iPSC cardiomyocyte model. In both evaluated parameters (Ca^2+^ oscillation frequency and Ca^2+^ peak width), the effects of the R(−) isomer were most potent. Considering that therapeutic oral doses of racemic HCQ produce sub‐micromolar free plasma concentrations of both enantiomers, the present data agree with previous findings of low arrhythmogenic events during HCQ therapy. However, caution should be exercised when racemic HCQ is prescribed at higher doses, especially in patients with heart conditions characterized by congenital long QT interval and an electrolyte imbalance. It can be speculated that in such situations the use of S(+)HCQ would produce a higher therapeutic window compared with the use of HCQ racemate.

## AUTHOR CONTRIBUTIONS

**Piotr K. Janicki** and **Victor Ruiz‐Velasco**: Conceptualized the study idea. **Piotr K. Janicki**, **Victor Ruiz‐Velasco**, **Amandeep Singh**, and **Arun K. Sharma**: Designed the study protocols; **Piotr K. Janicki** and **Victor Ruiz‐Velasco**: Collected, analyzed, and interpreted data; and **Piotr K. Janicki**, **Victor Ruiz‐Velasco**, and **Arun K. Sharma**: Prepared the manuscript and figures. All authors approved the manuscript in its final version.

## FUNDING INFORMATION

This work was funded by a Research Allocation Panel grant from the Penn State College of Medicine, Department of Anesthesiology and Perioperative Medicine to PKJ and VR‐V.

## CONFLICT OF INTEREST STATEMENT

The authors declare no competing interests.

## Supporting information


Video S1.

Video S2.
